# *In situ* fluorescence yield soft X-ray absorption spectroscopy of electrochemical nickel deposition processes with and without ethylene glycol†

**DOI:** 10.1039/d2ra01050j

**Published:** 2022-04-05

**Authors:** Akinobu Yamaguchi, Naoya Akamatsu, Shunya Saegusa, Ryo Nakamura, Yuichi Utsumi, Masaru Kato, Ichizo Yagi, Tomoko Ishihara, Masaki Oura

**Affiliations:** Laboratory of Advanced Science and Technology for Industry, University of Hyogo 3-1-2 Kouto, Kamigori Hyogo 678-1205 Japan yamaguti@lasti.u-hyogo.ac.jp; RIKEN SPring-8 Center 1-1-1, Kouto Sayo Hyogo 679-5148 Japan; Faculty of Environmental Earth Science, Graduate School of Environmental Science, Hokkaido University N10W5, Kita-ku Sapporo Hokkaido 060-0810 Japan masaru.kato@ees.hokudai.ac.jp

## Abstract

The electrochemical Ni deposition at a platinum electrode was investigated in a plating nickel bath in the presence and absence of ethylene glycol (EG) using fluorescence yield soft X-ray absorption spectroscopy (FY-XAS) in the Ni L_2,3_-edge and O K-edge regions under potential control. At ≤+0.35 V *vs.* the reversible hydrogen electrode (RHE), the electrochemical Ni deposition was detected by the Ni L_2,3_-edge FY-XAS in the presence of EG whereas almost no such event was observed in the absence of EG. A drastic decrease of FY-XAS intensities in the O K-edge region was also observed in the presence of EG at >+0.35 V *vs.* RHE, suggesting that the nano-/micro-structured Ni deposition initiated by the removal of water molecules occurs on the Pt electrode. The complex formation of Ni^2+^ with EG and the adsorption of EG on the Ni surface could play an important role in the Ni deposition. This study demonstrates that the *in situ* FY-XAS is a powerful and surface-sensitive technique to understand (electro)chemical reactions including polyol synthesis and electrocatalysis at solid–liquid interfaces.

## Abstract 2

Schematic drawing of electrochemical reactions of the Pt-coated SiC electrode, which separates the vacuum and the solution containing Ni^2+^ and ethylene glycol, in our spectro-electrochemical setup for the FY-XAS.

## Graphical abstract

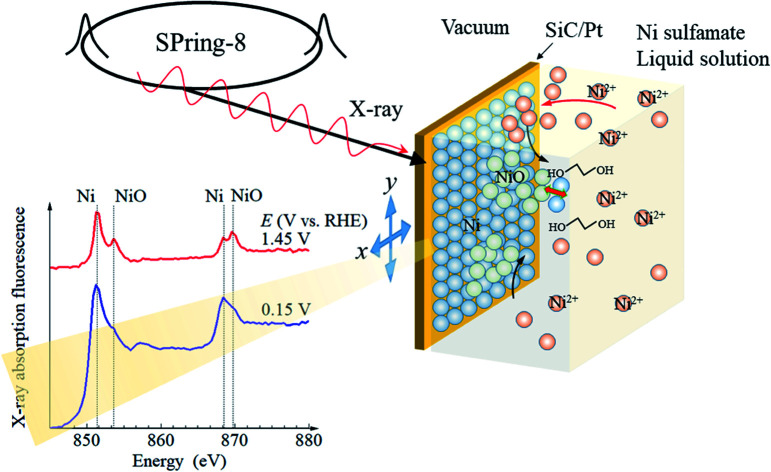

## Introduction

Electrochemical reactions including electroplating, electrodeposition, and electrocatalysis proceed at electrode–solution interfaces ranging up to several micrometers, where electric double layers are formed and modulate the interfacial reaction dynamics. Such interfacial reactions can also occur at solid–liquid interfaces in various research fields such as fuel cells, catalysis, and crystal growth and have been investigated using surface-sensitive techniques. For electrochemical reactions, many surface-sensitive techniques including an electrochemical quartz crystal microbalance (EQCM),^1,2^ surface-enhanced Raman scattering (SERS),^3,4^ surface-enhanced infrared absorption spectroscopy (SEIRAS),^5–8^ and surface X-ray scattering (SXS)^9^ have been used. For example, EQCM provides information on the amount of metal nanoparticles during electrochemical metal deposition but not on the electronic state of the metal deposited. Vibrational techniques of SERS and SEIRAS allow us to track bond formation and cleavage of metal oxides and organic molecules but not metal deposition.

*In situ* X-ray absorption spectroscopy (XAS) using soft X-rays is an emerging surface-sensitive technique particularly to understanding the electronic state of target elements including not only metals but light elements such as C, N, and O during electrochemical reactions at the solid–liquid interface. *In situ* XAS using soft X-rays enables us to track metal deposition and dissolution processes at the electrode interface and understand the electronic states of either metal or oxygen species on the electrode under potential control, where carefully designed spectro-electrochemical flow cells should be used depending on the XAS data acquisition method.^10–16^

Ni-based electrocatalysts have been used for sustainable energy production and storage including hydrogen evolution/oxidation and oxygen reduction/evolution reactions.^6,17–23^ Nickel can also be used to enhance the catalytic activity of noble metals including Pt by alloying^6,19,21,24^ or surface deposition,^25–27^ where the morphology, electronic structure, and/or interfacial charges of Pt modulated by Ni enable the catalytic activity enhancement. The electrochemical deposition of Ni on Pt would provide an excellent platform to understand the role of the Ni/Pt interface for electrocatalytic enhancements observed for Pt–Ni alloy electrocatalysts and the formation process of the Ni/Pt interface because the nucleation and growth process of Ni on metal substrates can be precisely controlled by potentials.^25^*In situ* XAS spectroscopy using soft X-rays would be suitable for tracking electrochemical events for the electrochemical deposition of Ni on Pt to form the Ni/Pt interface involving the change of chemical states on Ni and oxygen that can be found in solvents such as water.

In this work, we tracked the electrochemical deposition of Ni on a Pt electrode in plating solutions containing nickel sulfamate in the presence and absence of an organic additive of ethylene glycol (EG) by *in situ* photon-in/photon-out fluorescence yield XAS (FY-XAS) under electrochemical conditions. Nickel sulfamate solutions have been widely used for Ni electroplating because of low stress in the Ni deposit, high current densities suitable for the increase in production potential, and high ductility of the Ni deposit.^28,29^ Tracking the electrochemical Ni deposition process on the Pt electrode by *in situ* FY-XAS in the O K-edge and Ni L_2,3_-edge regions allows us to understand the formation mechanism of the Ni/Pt interface for Pt–Ni alloy electrocatalysts as well as the effect of EG in Ni electroplating on the crystallographic texture, grain size, mechanical properties and corrosion resistance of electrodeposited Ni.^25,29–32^

## Experimental methods

### Materials

Nickel sulfamate Ni(SO_3_NH_2_)_2_ (>98%), sodium sulfamate NaSO_3_NH_2_ (>98%) and ethylene glycol (EG, >99.5%) were purchased from FUJIFILM Wako Pure Chemical Corporation and then used without further purification.

### FY-XAS measurements using a spectro-electrochemical flow cell

**Fig. 1:**
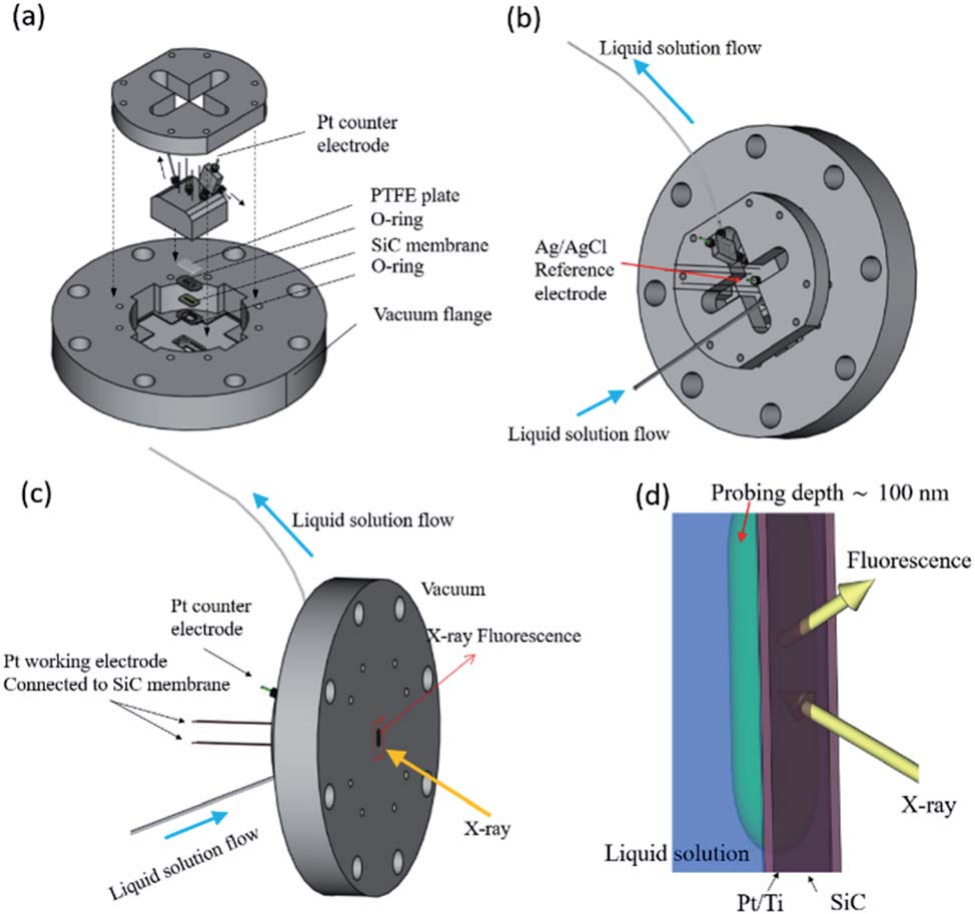
(a) Schematic representation of the spectro-electrochemical flow cell for photon-in/photon-out soft X-ray spectroscopy.^11^ The cell consists of a working electrode (a Pt thin film deposited on a SiC window), a Pt counter electrode, and a Ag|AgCl reference electrode in a 3 M KCl aqueous solution. Views of the cell are shown from (b) the atmospheric side and (c) the vacuum side. (d) Enlarged schematic representation of the interface between the solution and the Pt/SiC window, which can be found in the rectangle in red shown in (c).

FY-XAS experiments were performed at BL17SU beamline in SPring-8.^33,34^ The photon-in/photon-out FY-XAS spectra were recorded using a 100 mm^2^ Si photodiode (IRD AXUV-100G, Opto Diode Corp., USA) placed in the vacuum chamber.^35^ The energy resolution is *E*/Δ*E* ∼ 2800 at 540 eV (∼2000 at 850 eV). A custom-made spectro-electrochemical flow cell with the three-electrode configuration was used for FY-XAS measurements (Fig. 1a–c).^11^ A Pt-coated SiC membrane with a Ti adhesive layer was used as the working electrode. The window on the Pt side faces the electrolyte solution and separates the solution and vacuum (Fig. 1d). Electrochemical reactions occur at the surface of the 15 nm-thick Pt deposited on the 150 nm-thick SiC membrane *via* the 3 nm-thick Ti adhesive layer (purchased from NTT-AT). A Pt wire electroplated with Pt black was used as the counter electrode. An Ag|AgCl (3 M KCl, Thermo Scientific 66EE009) electrode was used as the reference electrode.

In our experimental setup, soft X-rays come from the vacuum side and reach the Pt–electrolyte (solid–electrolyte) interface with penetration depths, allowing us to track electrochemical events at the electrode interface even under potential control. X-ray penetration depths in our experimental setup can be estimated to be approximately 300 nm at 550 eV in the O K-edge region and approximately 100 nm at ∼860 eV in the Ni L_2,3_-edge region, where the penetration depths are defined as the attenuation length at which the incident X-ray intensity decays to the (1/*e*)th (Napier's constant) of the initial value, assuming the attenuation of the window materials of the SiC membrane, Ti adhesion layer and Pt layer (see Fig. S1 and S2 in the ESI† for details.) A X-ray probing depth in Fig. 1d can also be estimated in the same way to be approximately 100 nm: the probing depth is defined as the attenuation length at which the signal intensity decays to the (1/*e*)th of the initial intensity from the outermost surface.

### Electrochemical measurements

A potentiostat (ECstat-101, EC-Frontier Co., Ltd., Japan) was used for *in situ* FY-XAS measurements. A potentiostat (Interface 1000T, Gamry Instruments, USA) was used for cyclic voltammetry measurements. Electrolyte solutions were degassed by N_2_ bubbling and introduced into the flow cell using an external liquid pump. Cyclic voltammograms (CVs) were recorded at a sweep rate of 0.05 V s^−1^. The potentials *vs.* Ag|AgCl (3 M KCl aq.), *E*_Ag|AgCl_, were converted to the potentials *vs.* the reversible hydrogen electrode (RHE), *E*_RHE_, using the following equation: *E*_RHE_ = *E*_Ag|AgCl_ + 0.205 + pH × 0.059.^35^ For recording CVs, electrolyte solutions of Ni(SO_3_NH_2_)_2_ (300 g L^−1^, 1.2 M) and NaSO_3_NH_2_ (142 g L^−1^, 1.2 M) were used. To investigate the polyol reaction characteristics, 1% v/v (1.6 × 10^−6^ M) EG was added to the solutions. The pHs of the Ni(SO_3_NH_2_)_2_ and NaSO_3_NH_2_ solutions were determined to be 5.83 and 9.03, respectively.

## Results and discussion

**Fig. 2:**
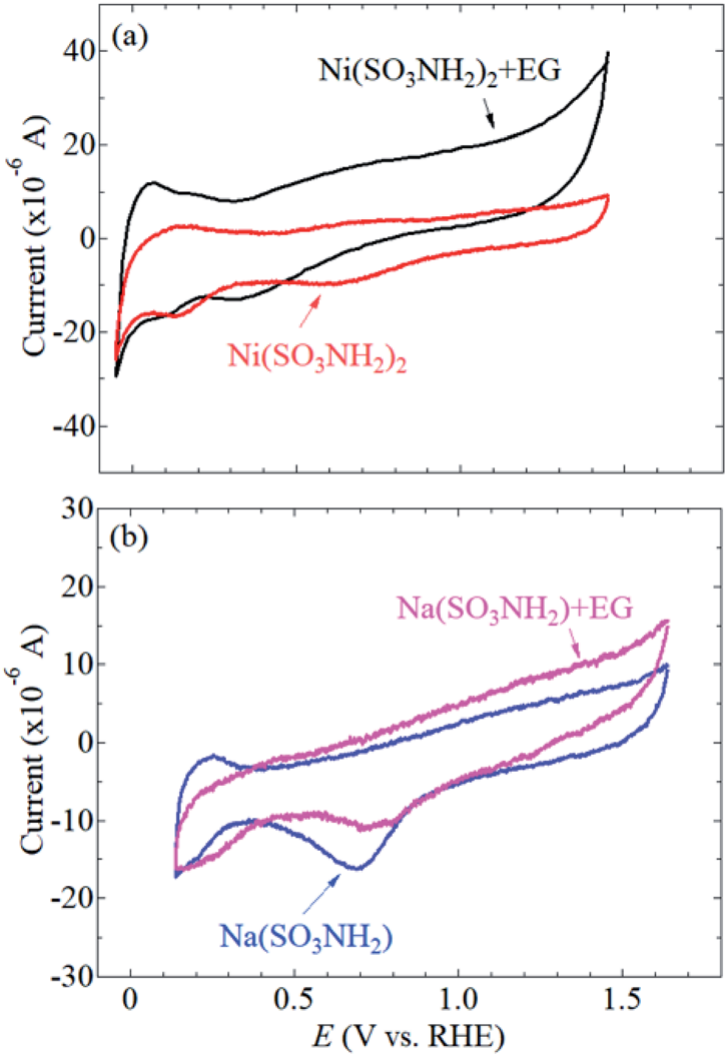
CVs of Pt/SiC electrodes in aqueous solutions containing (a) Ni(SO_3_NH_2_)_2_ and (b) NaSO_3_NH_2_ in the presence and absence of EG.

CVs of the Pt/SiC electrode were recorded in aqueous solutions containing Ni(SO_3_NH_2_)_2_ (Fig. 2a) or NaSO_3_NH_2_ (Fig. 2b) in the absence of EG. NaSO_3_NH_2_ was used as a redox-inactive counterpart for control experiments to understand the redox behavior of Ni^2+^ in solution. In the potential range between about +0.1 and +0.30 V *vs.* RHE, characteristic cathodic and anodic currents were observed in the NaSO_3_NH_2_ solution. These currents are associated with absorption and desorption of the underpotential deposition (upd) of hydrogen on platinum.^11,36–38^ A reduction wave at about +0.7 V *vs.* RHE was also observed and assigned to the re-reduction of Pt oxide. These results indicate that the surface of Pt was electrochemically active in the NaSO_3_NH_2_ solution. Even in the Ni(SO_3_NH_2_)_2_ solution, the characteristic cathodic and anodic currents of Pt were also observed. No obvious redox wave that originates from Ni^2+^ was observed in the absence of EG. This result is in good agreement with reported results that the Ni upd is known to be overlapped with the hydrogen upd on polycrystalline Pt.^39–42^

In the presence of EG in the Ni(SO_3_NH_2_)_2_ solution, a reduction wave was observed at about +0.35 V *vs.* RHE (Fig. 2a), indicating that the Ni upd clearly occurs on the Pt surface in the presence of EG. EG can be coordinated to transition metal cations including Ni^2+^ in solution.^43–45^ It is also known that the electrochemical deposition of NiO or Ni(OH)_2_ is enhanced by the complex formation of Ni^2+^, where bidentate ligands such as glycine and ethylenediamine are more effective than monodentate ligands of NH_3_ or OH_2_.^46–48^ Since EG is a bidentate ligand, the complex formation of Ni^2+^ with EG could enhance the electrochemical deposition of NiO or Ni(OH)_2_ on the Pt/SiC surface. Note that the reduction wave for the re-reduction of Pt oxide was shifted to more positive potentials in the presence of EG (Fig. 2b). This result indicates that EG could also be helpful for not only Ni upd but also the electrochemical reduction of the surface Pt oxide.

**Fig. 3:**
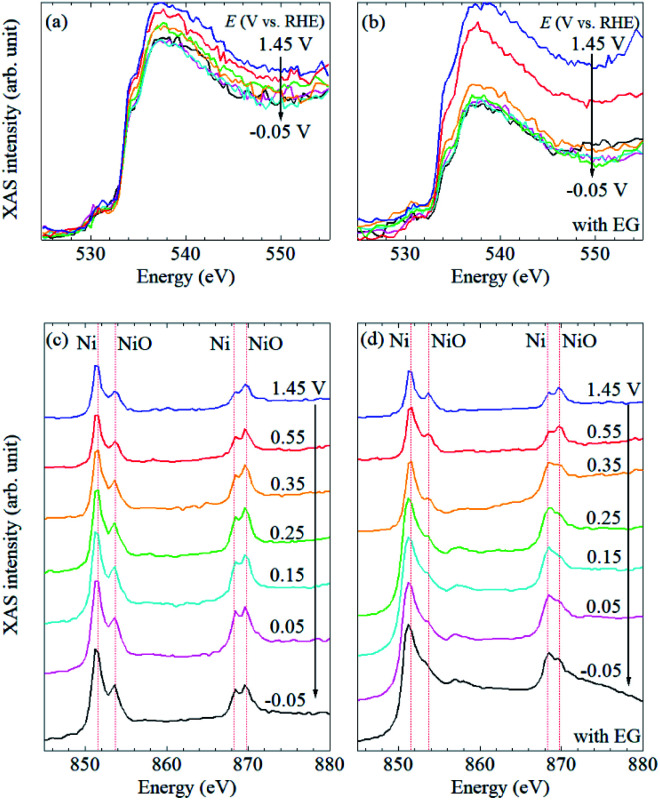
Potential-dependent FY-XAS data of Pt/SiC in the Ni(SO_3_NH_2_)_2_ solution in the O K-edge in the absence (a) and presence (b) of EG and in the Ni L_2,3_-edge region in the absence (c) and the presence (d) of EG. The arrows indicate the direction of the potential changes.

To understand the electrochemical events at the liquid–electrode interface, potential-dependent FY-XAS data of the Pt/SiC electrode were collected in the Ni(SO_3_NH_2_)_2_ solution in the absence or presence of EG in the O K-edge (Fig. 3a and b) and Ni L_2,3_-edge regions (Fig. 3c and d). The applied potentials were stepped in the positive or negative direction. In both cases, the same spectra were obtained, (Fig. S3†), indicating that the spectral changes are reversible and reproduced by potential control.

In the Ni L_2,3_-edge region, mainly four peaks were observed at about 851 and 853 eV for the Ni 2p_3/2_ → 3d transition and approximately 869 and 870 eV for the Ni 2p_3/2_ → 3d transition. For further assignments of these transitions to the exact 3d orbital, careful experiments in the magnetic field and theoretical calculations are needed. The peaks around 851 and 869 eV, which are found at lower energies in each transition, can be associated with metallic Ni^35,49^ whereas the peaks around 853 and 870 eV, which are found at higher energies in each transition, can be assigned to NiO.^16,50,51^ Thus, *in situ* FY-XAS using our spectro-electrochemical flow cell allows us to detect the deposition of Ni and NiO on the Pt electrode.

In the O K-edge region, two peaks were observed in the vicinity of 535 and 537 eV (Fig. 3a and b). There is no clear difference between the spectra in the presence and absence of EG, and the spectral shape is quite similar to that of pure water.^11^ No obvious peak was observed at 532 eV (Fig. S4†), at which a peak is observed for NiO or β-Ni(OH)_2_,^12^ even though the presence of NiO was confirmed by Ni L_2,3_-edge XAS data, as mentioned above. It seems that signal-to-noise ratios in the O K-edge XAS are not high enough to observe the peak at 532 eV. Thus, the spectral changes in the O K-edge region mainly originate from changes involving H_2_O molecules at the liquid–electrode interface. Since the spectral changes were completed at +0.35 V *vs.* RHE even in the presence of EG (Fig. 3b), it seems that the Pt electrode surface was not fully covered with the Ni or NiO deposit and exposed to the electrolyte solution at >+0.35 V *vs.* RHE.

The potential dependence of the peak intensities in the Ni L_2,3_-edge region was observed in the presence of EG (Fig. 3d) whereas there is no obvious spectral change in the absence of EG (Fig. 3c). In the presence of EG, peak intensity ratios at 851/853 eV and 869/870 eV increased at more negative potentials (Fig. S5†). Since these peak ratios can be correlated with the Ni/NiO ratio based on our peak assignments, the amount of metallic Ni increased at negative potentials. A broad peak was also observed around 857 eV at ≤0.35 V *vs.* RHE (Fig. 3d). Although β-Ni(OH)_2_ is reported to show a peak around 859 eV,^16^ the broad peak observed at about 857 eV could be assigned to the satellite peak of metallic Ni.^36^ It is highly likely that the peak was pronounced at ≤0.35 V *vs.* RHE by the deposition and/or nucleation of metallic Ni nano-/micro-structured particles on the Pt electrode. The increase of this broad peak is coupled with the drastic decrease of O K-edge FY-XAS intensities at >533 eV in the presence of EG (Fig. 3b). Thus, the removal of water molecules from the Pt surface mainly happens at >0.35 V *vs.* RHE and the Ni deposition starts at the Pt surface at ≤ 0.35 V *vs.* RHE.

Note that the drastic increase of the baseline intensities was also observed in the Ni L_2,3_-edge spectra in the presence of EG (Fig. 3d). Such similar spectral changes were reported in depth-resolved Ni L_2,3_-edge XAS spectra of O/Ni/Cu(100), where the inner Ni layers showed a broad peak at about 857 eV and higher baseline than the surface Ni.^35^ Since the photon-in and photon-out angle was kept during our all measurements, the increase of the baseline intensity observed in the Ni L_2,3_-edge spectra at ≤0.35 V *vs.* RHE in the presence of EG can be interpreted as the increase of the film thickness of deposited metallic Ni nano/micro-particles on the Pt/SiC electrode. In our experimental setup, the X-ray probing depth can be estimated to be about 100 nm (Fig. 1d) and O K-edge FY-XAS signals of water molecules were detected (Fig. 3b). Thus, the film thickness of the nickel deposited onto the Pt electrode can be estimated to be <100 nm.

**Fig. 4:**
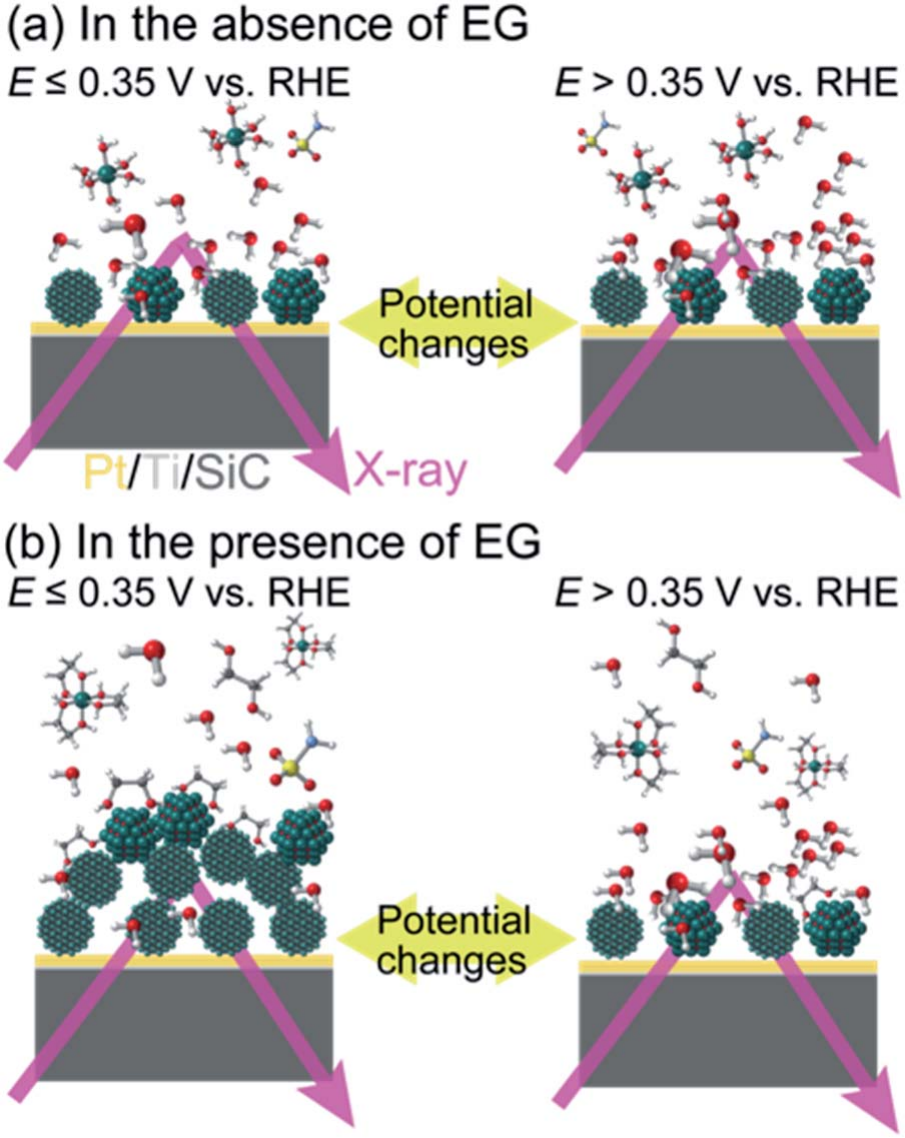
Schematic representation of proposed nano-/micro-structured Ni deposition and dissolution at the liquid–electrode interface (a) in the absence and (b) presence of EG. Spheres in green, red, gray, white, yellow, blue indicate nickel, oxygen, carbon, hydrogen, sulfur and nitrogen atoms, respectively.

Based on our FY-XAS experimental results, electrochemical events at the liquid–electrode interface in the presence and absence of EG are summarized in Fig. 4. In the absence of EG, almost no spectral difference was observed in the Ni L_2,3_-edge region, indicating that no obvious structural change was detected in our setup. In the presence of EG, the removal of water molecules from the electrode surface occurs at >+0.35 V *vs.* RHE in the negative-going potential steps, revealed by O K-edge FY-XAS results. The electrochemical deposition and/or nucleation of metallic Ni nano-/micro-structured particles on the Pt electrode occurred at ≤+0.35 V *vs.* RHE. This Ni deposition process can be facilitated by ligand exchange reactions of Ni^II^–EG complexes and/or stabilization of the Ni particles adsorbed by EG on the surface.^52,53^ Since the potential dependence of the Ni L_2,3_-edge spectra is reversible, the electrochemical re-oxidation and dissolution of Ni particles occurred at >+0.35 V *vs.* RHE.

## Conclusions

The electrochemical Ni deposition on the Pt/SiC electrode in the electrolyte solutions containing nickel sulfamate in the presence and absence of EG was tracked by *in situ* Ni L_2,3_-edge and O K-edge FY-XAS. In the presence of EG, water molecules are removed from the electrode surface at >+0.35 V *vs.* RHE and nano-/micro-structured Ni is electrochemically deposited at ≤+0.35 V *vs.* RHE. The Ni^II^ complex formation and surface adsorption of EG likely play a crucial role in the electrochemical Ni deposition. Our study demonstrates that *in situ* FY-XAS can be widely used to understand the formation process of nano-/micro-structured Ni at the solid–liquid interface in polyol reactions^52^ and electroplating, and the redox behavior of Ni involving (electro)catalytic reactions.

For widespread applications of *in situ* FY-XAS techniques for electrochemical systems, further *in situ* FY-XAS studies are needed: for example, effects of X-ray radiolysis-induced photochemical reactions on the metal deposition. Metals and/or metal oxides are deposited by X-ray radiolysis-induced photochemical reactions and are known to highly depend on the electrode materials rather than the electrolyte.^54–56^ Currently, *in situ* FY-XAS studies using different metal electrode substrates under different experimental conditions are underway in our group.

## Author contributions

AY proposed and directed this study. MK and IY designed the electrochemical cell. TI and MO prepared the synchrotron radiation experimental setup. AY, NA, SS, RN, YU, TI and MO performed synchrotron radiation and electrochemical experiments. AY and MK wrote the manuscript.

## Conflicts of interest

There are no conflicts to declare.

## Supplementary Material

RA-012-D2RA01050J-s001

## References

[cit1] Buttry D. A., Ward M. D. (1992). Chem. Rev..

[cit2] Shpigel N., Levi M. D., Sigalov S., Daikhin L., Aurbach D. (2018). Acc. Chem. Res..

[cit3] Li J. F., Huang Y. F., Ding Y., Yang Z. L., Li S. B., Zhou X. S., Fan F. R., Zhang W., Zhou Z. Y., Wu D. Y., Ren B., Wang Z. L., Tian Z. Q. (2010). Nature.

[cit4] Ikeda K., Takahashi K., Masuda T., Kobori H., Kanehara M., Teranishi T., Uosaki K. (2012). J. Phys. Chem. C.

[cit5] Kato M., Nakagawa S., Tosha T., Shiro Y., Masuda Y., Nakata K., Yagi I. (2018). J. Phys. Chem. Lett..

[cit6] Kato M., Ogura K., Nakagawa S., Tokuda S., Takahashi K., Nakamura T., Yagi I. (2018). ACS Omega.

[cit7] Kato M., Masuda Y., Yoshida N., Tosha T., Shiro Y., Yagi I. (2021). Electrochim. Acta.

[cit8] Yang X., Sun Z., Low T., Hu H., Guo X., García de Abajo F. J., Avouris P., Dai Q. (2018). Adv. Mater..

[cit9] Yasuda S., Tamura K., Kato M., Asaoka H., Yagi I. (2021). J. Phys. Chem. C.

[cit10] Velasco-Velez J.-J., Wu C. H., Pascal T. A., Wan L. F., Guo J., Prendergast D., Salmeron M. (2014). Science.

[cit11] Ishihara T., Tokushima T., Horikawa Y., Kato M., Yagi I. (2017). Rev. Sci. Instrum..

[cit12] Yoshida M., Mitsutomi Y., Mineo T., Nagasaka M., Yuzawa H., Kosugi N., Kondoh H. (2015). J. Phys. Chem. C.

[cit13] Nagasaka M., Yuzawa H., Horigome T., Hitchcock A. P., Kosugi N. (2013). J. Phys. Chem. C.

[cit14] Bordiga S., Groppo E., Agostini G., van Bokhoven J. A., Lamberti C. (2013). Chem. Rev..

[cit15] Timoshenko J., Roldan Cuenya B. (2021). Chem. Rev..

[cit16] Al Samarai M., Hahn A. W., Beheshti Askari A., Cui Y.-T., Yamazoe K., Miyawaki J., Harada Y., Rüdiger O., DeBeer S. (2019). ACS Appl. Mater. Interfaces.

[cit17] Oshchepkov A. G., Braesch G., Bonnefont A., Savinova E. R., Chatenet M. (2020). ACS Catal..

[cit18] Vij V., Sultan S., Harzandi A. M., Meena A., Tiwari J. N., Lee W.-G., Yoon T., Kim K. S. (2017). ACS Catal..

[cit19] Kato M., Nakahoshiba R., Ogura K., Tokuda S., Yasuda S., Higashi K., Uruga T., Uemura Y., Yagi I. (2020). ACS Appl. Energy Mater..

[cit20] Luo F., Wagner S., Onishi I., Selve S., Li S., Ju W., Wang H., Steinberg J., Thomas A., Kramm U. I., Strasser P. (2021). Chem. Sci..

[cit21] Chen C., Kang Y. J., Huo Z. Y., Zhu Z. W., Huang W. Y., Xin H. L. L., Snyder J. D., Li D. G., Herron J. A., Mavrikakis M., Chi M. F., More K. L., Li Y. D., Markovic N. M., Somorjai G. A., Yang P. D., Stamenkovic V. R. (2014). Science.

[cit22] Reuillard B., Blanco M., Calvillo L., Coutard N., Ghedjatti A., Chenevier P., Agnoli S., Otyepka M., Granozzi G., Artero V. (2020). ACS Appl. Mater. Interfaces.

[cit23] Zeng H., Chen S., Jin Y. Q., Li J., Song J., Le Z., Liang G., Zhang H., Xie F., Chen J., Jin Y., Chen X., Meng H. (2020). ACS Energy Lett..

[cit24] Stamenkovic V. R., Fowler B., Mun B. S., Wang G., Ross P. N., Lucas C. A., Marković N. M. (2007). Science.

[cit25] Xu C., Chen P., Hu B., Xiang Q., Cen Y., Hu B., Liu L., Liu Y., Yu D., Chen C. (2020). CrystEngComm.

[cit26] Danilovic N., Subbaraman R., Strmcnik D., Chang K.-C., Paulikas A. P., Stamenkovic V. R., Markovic N. M. (2012). Angew. Chem., Int. Ed..

[cit27] Subbaraman R., Tripkovic D., Chang K.-C., Strmcnik D., Paulikas A. P., Hirunsit P., Chan M., Greeley J., Stamenkovic V., Markovic N. M. (2012). Nat. Mater..

[cit28] Baudrand D. (1996). Met. Finish..

[cit29] Mohanty U. S., Tripathy B. C., Singh P., Keshavarz A., Iglauer S. (2019). J. Appl. Electrochem..

[cit30] Wu X.-L., Ma E. (2006). Appl. Phys. Lett..

[cit31] Pathak S., Guinard M., Vernooij M. G. C., Cousin B., Wang Z., Michler J., Philippe L. (2011). Surf. Coat. Technol..

[cit32] Godon A., Creus J., Feaugas X., Conforto E., Pichon L., Armand C., Savall C. (2011). Mater. Charact..

[cit33] Ohashi H., Senba Y., Kishimoto H., Miura T., Ishiguro E., Takeuchi T., Oura M., Shirasawa K., Tanaka T., Takeuchi M., Takeshita K., Goto S., Takahashi S., Aoyagi H., Sano M., Furukawa Y., Ohata T., Matsushita T., Ishizawa Y., Taniguchi S., Asano Y., Harada Y., Tokushima T., Horiba K., Kitamura H., Ishikawa T., Shin S. (2007). AIP Conf. Proc..

[cit34] Senba Y., Ohashi H., Kishimoto H., Miura T., Goto S., Shin S., Shintake T., Ishikawa T. (2007). AIP Conf. Proc..

[cit35] Amemiya K. (2012). Phys. Chem. Chem. Phys..

[cit36] Clavilier J., Faure R., Guinet G., Durand R. (1980). J. Electroanal. Chem. Interfacial Electrochem..

[cit37] Kato M., Okui M., Taguchi S., Yagi I. (2017). J. Electroanal. Chem..

[cit38] Herrero E., Buller L. J., Abruña H. D. (2001). Chem. Rev..

[cit39] Franaszczuk K., Sobkowski J. (1988). Surf. Sci..

[cit40] El-Shafei A. A. (1998). J. Electroanal. Chem..

[cit41] Sarabia F. J., Climent V., Feliu J. M. (2018). J. Electroanal. Chem..

[cit42] Chatenet M., Faure R., Soldo-Olivier Y. (2005). J. Electroanal. Chem..

[cit43] Teichert J., Ruck M. (2019). Eur. J. Inorg. Chem..

[cit44] Nylander L. R., Pavkovic S. F. (1970). Inorg. Chem..

[cit45] Antti B. M. (1975). Acta Chem. Scand., Ser. A.

[cit46] Singh A., Chang S. L. Y., Hocking R. K., Bach U., Spiccia L. (2013). Energy Environ. Sci..

[cit47] Yoshida M., Onishi S., Mitsutomi Y., Yamamoto F., Nagasaka M., Yuzawa H., Kosugi N., Kondoh H. (2017). J. Phys. Chem. C.

[cit48] Wang D., Ghirlanda G., Allen J. P. (2014). J. Am. Chem. Soc..

[cit49] Nakamura R., Saegusa S., Akamatsu N., Yamada K., Ogasawara T., Oura M., Ohkochi T., Yamaguchi A. (2021). Jpn. J. Appl. Phys..

[cit50] Stöhr J., Scholl A., Regan T. J., Anders S., Lüning J., Scheinfein M. R., Padmore H. A., White R. L. (1999). Phys. Rev. Lett..

[cit51] Ishii H., Ishiwata Y., Eguchi R., Harada Y., Watanabe M., Chainani A., Shin S. (2001). J. Phys. Soc. Jpn..

[cit52] Dong H., Chen Y. C., Feldmann C. (2015). Green Chem..

[cit53] Carroll K. J., Reveles J. U., Shultz M. D., Khanna S. N., Carpenter E. E. (2011). J. Phys. Chem. C.

[cit54] Yamaguchi A., Sakurai I., Okada I., Izumi H., Ishihara M., Fukuoka T., Suzuki S., Utsumi Y. (2020). J. Synchrotron Radiat..

[cit55] Saegusa S., Sakurai I., Okada I., Fukuoka T., Suzuki S., Utsumi Y., Yamaguchi A. (2019). Trans. Jpn. Inst. Electron. Packag..

[cit56] Yamaguchi A., Okada I., Sakurai I., Izumi H., Ishihara M., Fukuoka T., Suzuki S., Elphick K., Jackson E., Hirohata A., Utsumi Y. (2019). J. Synchrotron Radiat..

