# Supplementary material for: In situ fluorescence yield soft X-ray absorption spectroscopy of electrochemical nickel deposition processes with and without ethylene glycol
Source: RSC Adv. 2022 Apr 5;12(17):10425–30. doi: 10.1039/d2ra01050j (PMC8982338; doi:10.1039/d2ra01050j)
Supplement: RA-012-D2RA01050J-s001 [file RA-012-D2RA01050J-s001.pdf]

Electronic Supplementary Material (ESI) for RSC Advances.
This journal is © The Royal Society of Chemistry 2022

Electronic Supplementary Information for

In Situ Fluorescence Yield Soft X-ray Absorption Spectroscopy of Electrochemical Nickel Deposition Processes with and without Ethylene Glycol

Akinobu Yamaguchi,*[a,b] Naoya Akamatsu,[a,b] Shunya Saegusa,[a,b] Ryo Nakamura,[a,b] Yuichi Utsumi,[a] Masaru Kato,*[b,c] Ichizo Yagi,[b,c] Tomoko Ishihara,[b] Masaki Oura[b]

[a] Laboratory of Advanced Science and Technology for Industry, University of Hyogo, 3-1-2 Kouto, Kamigori, Hyogo 678-1205, Japan

[b] RIKEN SPring-8 Center, 1-1-1, Kouto, Sayo, Hyogo 679-5148, Japan

[c] Faculty of Environmental Earth Science and Graduate School of Environmental Science, Hokkaido University, N10W5, Kita-ku, Sapporo, Hokkaido 060-0810, Japan

*Contact address: yamaguti@lasti.u-hyogo.ac.jp (AY) & masaru.kato@ees.hokudai.ac.jp (MK)

CONTENTS

S1. Evaluation of X-ray transmission as a function of energy.

S2. Estimation of thickness dependence of X-ray transmittance at O-K and Ni-L edges.

S3. Potential dependence of FY-XAS data of Ni $L_{2,3}$-edges region in the positive direction

S4. Potential dependence of FY-XAS data of O K-edge region magnified around 532 eV

S1. Evaluation of X-ray transmission as a function of energy.

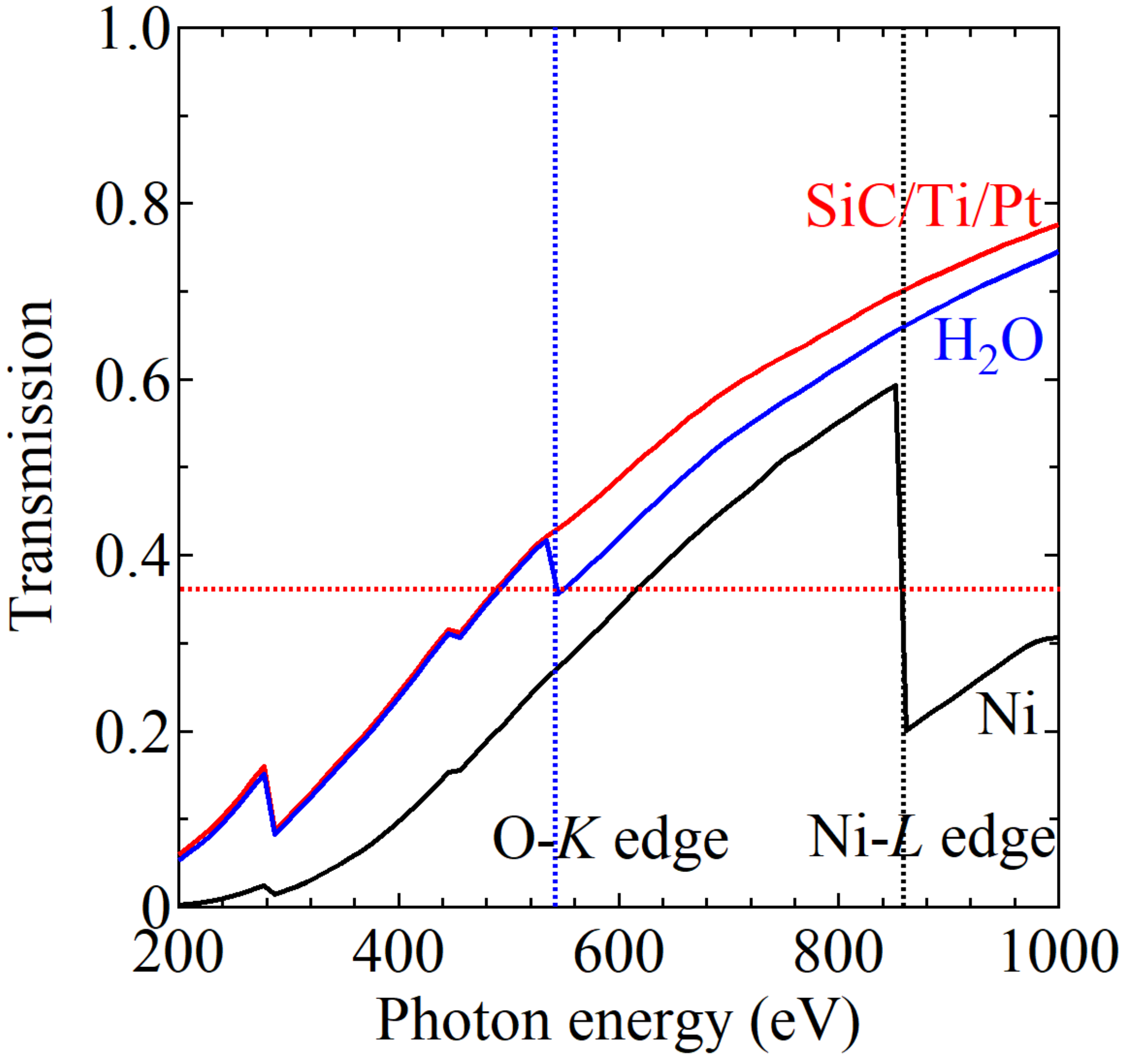

Fig. S1

Plots of calculated X-ray transmittances of SiC/Ti/Pt window (the solid line in red), 100-nm-thick water through the window (the solid line in blue), and 100-nm-thick Ni film through the water (the solid line in black) as a function of photon energy. The SiC/Ti/Pt window consists of the 15-nm-thick Pt deposited on the 150-nm-thick SiC membrane via the 3-nm-thick Ti adhesive layer. The calculations were performed using parameters available from the website of the Center of X-ray Optics (https://henke.lbl.gov/optical_constants/). The dotted lines in red indicate the X-ray transmittances at the attenuation length at which the incident X-ray intensity decays to the (1/*e*)th of the initial value.

S2. Estimation of thickness dependence of X-ray transmittance at O-K and Ni-L edges.

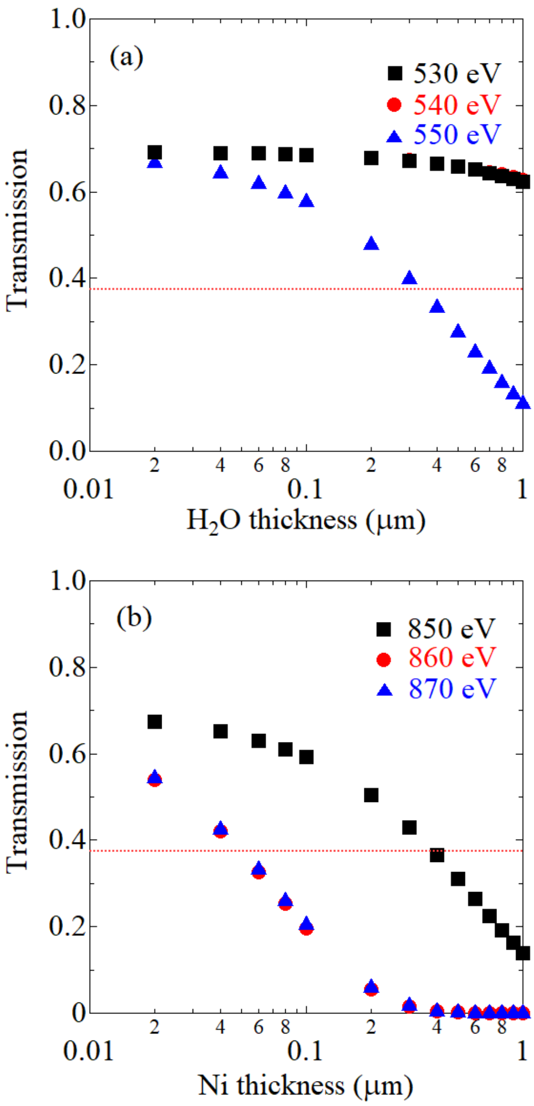

Fig. S2

Thickness dependence of calculated X-ray transmittances for (a) water and (b) a Ni film after passing through the window of 150-nm-thick-SiC membrane, 3-nm-thick Ti adhesion layer, and 15-nm-thick Pt electrode. The energies of X-ray were set to be 530, 540, and 550 eV in the oxygen absorption edge region for water and to be 830, 840, and 850 eV for the Ni absorption edge. The dotted lines in red indicate the X-ray transmittances at the attenuation length at which the incident X-ray intensity decays to the (1/*e*)th of the initial value.

S3. Potential dependence of FY-XAS data of Ni $L_{2,3}$-edge region in the positive direction

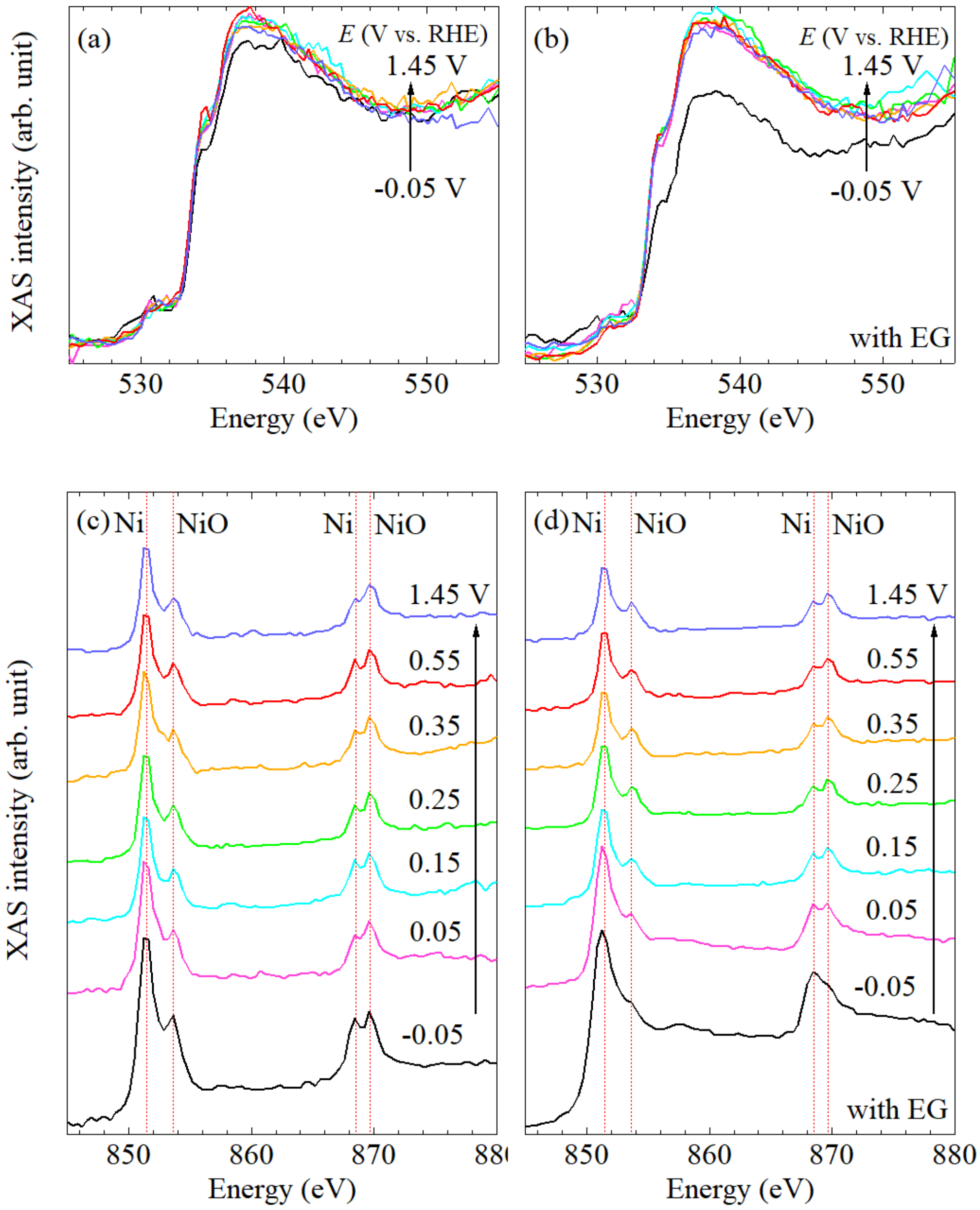

Fig. S3

Potential-dependent FY-XAS data of Pt/SiC in the $Ni(SO_3NH_2)_2$ solution in the O *K*-edge in the absence (a) and presence (b) of EG and in the Ni $L_{2,3}$-edge region in the absence (c) and the presence (d) of EG. The arrows indicate the direction of the potential changes.

S4. Potential dependence of FY-XAS data of O *K*-edge region magnified around 532 eV

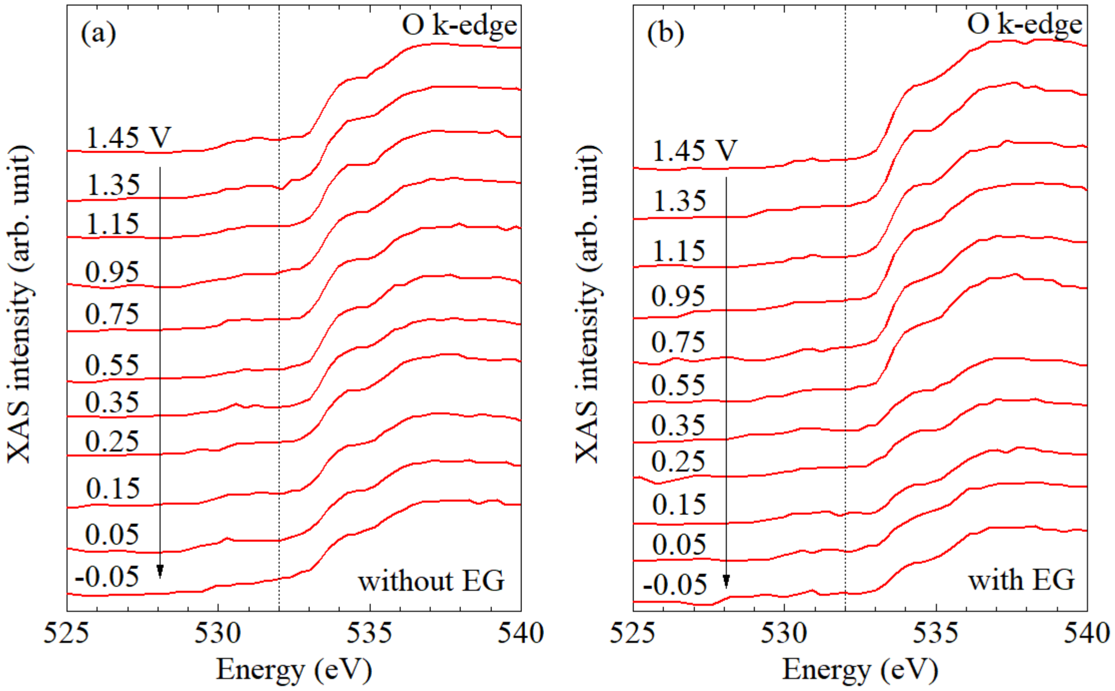

Fig. S4

Potential-dependent FY-XAS data of Pt/SiC in the $Ni(SO_3NH_2)_2$ solution in the O *K*-edge in the absence (a) and presence (b) of EG and in the O *K*-edge region in the absence (c) and the presence (d) of EG. The arrows indicate the direction of the potential changes.

S5. Potential dependence of peak intensity ratios in the Ni $L_{2,3}$-edge region

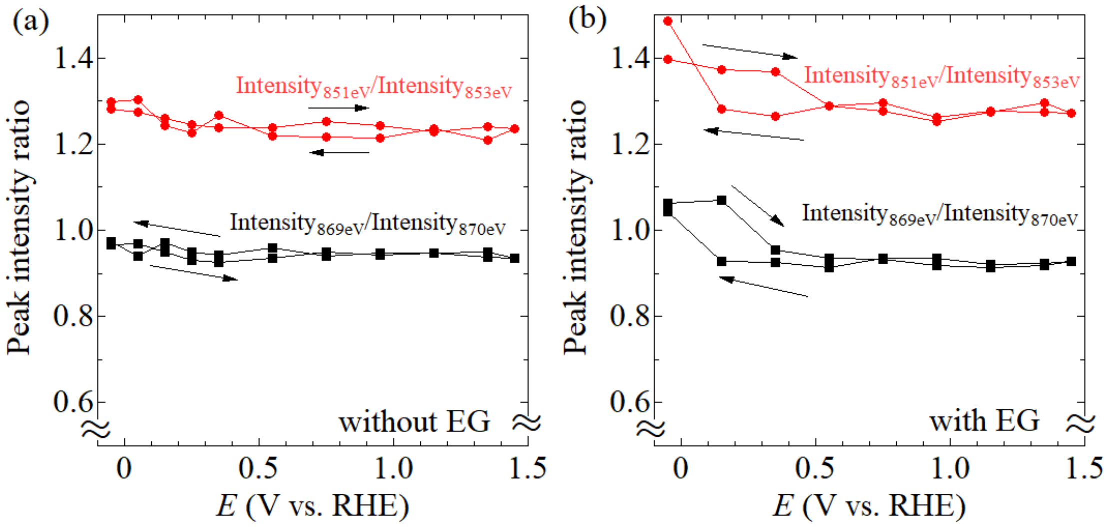

Fig. S5

Potential dependence of peak intensities ratios at 851/853 eV (the filled circles in red) and 869/870 eV (the filled squares in black) (a) in the absence and (b) presence of EG. These plots are based on the data in **Fig. 3c** and **3d** in the main text and **Fig. S3c** and **S3d**. The arrows indicate that the direction of the potential changes.
